# Binding selectivity analysis of AURKs inhibitors through molecular dynamics simulation studies

**DOI:** 10.1371/journal.pone.0295741

**Published:** 2023-12-19

**Authors:** Rima D. Alharthy, Ghulam Fatima, Numan Yousaf, Muhammad Shaheen Iqbal, Sadia Sattar, Abdullah R. Alanzi, Ijaz Ali, Muhammad Muddassar

**Affiliations:** 1 Department of Chemistry, Science and Arts College, King Abdulaziz University, Jeddah, Saudi Arabia; 2 Department of Biosciences, COMSATS University Islamabad, Islamabad, Pakistan; 3 Department of Pharmacognosy, College of Pharmacy, King Saud University, Riyadh, Saudi Arabia; 4 Centre for Applied Mathematics and Bioinformatics (CAMB), Gulf University for Science and Technology, Hawally, Kuwait; Saveetha University - Poonamallee Campus: SIMATS Deemed University, INDIA

## Abstract

Aurora kinases (AURKs) have been identified as promising biological targets for the treatment of cancer. In this study, molecular dynamics simulations were employed to investigate the binding selectivity of three inhibitors (HPM, MPY, and VX6) towards AURKA and AURKB by predicting their binding free energies. The results show that the inhibitors HPM, MPY, and VX6 have more favorable interactions with AURKB as compared to AURKA. The binding energy decomposition analysis revealed that four common residue pairs (L139, L83), (V147, V91), (L210, L154), and (L263, L207) showed significant binding energies with HPM, MPY, and VX6, hence responsible for the binding selectivity of AURKA and AURKB to the inhibitors. The MD trajectory analysis also revealed that the inhibitors affect the dynamic flexibility of protein structure, which is also responsible for the partial selectivity of HPM, MPY, and VX6 towards AURKA and AURKB. As expected, this study provides useful insights for the design of potential inhibitors with high selectivity for AURKA and AURKB.

## 1. Introduction

The Aurora kinase group is composed of serine/threonine kinases, known as Aurora kinase A (AURKA), Aurora kinase B (AURKB), and Aurora kinase C (AURKC) [1]. AURKs serve an important role in controlling the cell cycle, with AURKA and AURKB being particularly important during mitosis [1], while AURKC, is crucial for gametogenesis [2]. The kinase domain of AURKs, containing three distinct domains, is highly homologous across all of its members [3]. But the N-terminal region’s sequence vary [3]. The location and spatiotemporal expression of AURKs clearly characterize their functional roles [4]. According to study, the overexpression of AURKs in malignancies results in genomic instability and aneuploidy [5] which causes the development, invasion, and spread of a tumor. Several studies emphasize the significance of AURKA in cancer treatment after extensive research into its functions [6].

AURKA and AURKB are two essential members of the serine/threonine kinases group [1, 7], with AURKA being associated with mitotic commitment, spindle construction, spindle maintenance, and centrosome function [8, 9]. TPX2 plays a crucial role in localizing AURKA to the mitotic spindle by binding and activating it. [10] The association between AURKA and TPX2 relies on the presence of glycine 198 (G198) in the catalytic domain of AURKA [10]. Additionally, the interaction between protein phosphatase 1 and AURKA, regulated by phosphorylation during mitosis, is vital for proper chromosomal segregation [11]. On the other hand, AURKB, encoded by the AURKB gene on chromosome 17, also plays a critical role in regulating the cell cycle. Both AURKA and AURKB phosphorylate histone H3, which is essential for chromosomal segregation during cell division [12]. AURKB is linked to the Chromosomal Passenger Complex, consisting of Survivin, Borealin, and INCENP, which plays a key role in various aspects of mitosis, including chromosome alignment, cytokinesis, and segregation.

Two significant regulators of cell division, aurora kinases A and B, have very similar amino acid sequences. The N-terminal domains, protein kinase domains, and C-terminal domains of Aurora A and B have an impressive conservation rate, with their catalytic domains being 71% identical [3]. Furthermore, the 26 residues around the ATP-binding active regions of both kinases are similar: In Aurora A, L215, T217, and R220, and in Aurora B, R159, E161, and K164, are the only residues differentiating their ATP-binding sites [13]. Despite these similarities, Aurora A and Aurora B have different chromosomal affiliations: Aurora A is linked to chromosome 20q13.2, whereas Aurora B is linked to chromosome 17p13.1 [14]. The average fraction of similar amino acids within the vertebrate Aurora A and B families is significantly larger (0.84 ± 0.5) than within either family alone, suggesting recent vertebrate evolution [15]. The high conservation rate is essential in relation to the distinctive pairing of substrates and inhibitors. These kinases interact with diverse substrates and subcellular localizations with minimal sequence change, despite their dissimilar structures and motifs. This highlights the essential functions that both kinds of kinases carry out in regulating cell division.Many studies have been reported that overexpression of AURKs are responsible in variety of human cancers, and the mutations in Aurora kinases have been identified in a variety of somatic cancer samples, that includes lung cancer, and melanoma [16], this suggests that the function of Aurora kinases in cell transformation and oncogenesis is crucial. In recent decades, there has been increased research on the role of these potentially oncogenic proteins in tumor growth.

The carboxyl terminus catalytic domain of AURKA and AURKB share approximately 70% similarity. Both kinases are essential for mitotic progression, but they have distinct localizations and roles. To investigate the reason for the difference between AURKA and AURKB, studies have used paired shRNA suppression with overexpression of Aurora mutants. Results showed that when the catalytic domain residue, glycine 198 is replaced with asparagine to mimetic the aligned asparagine 142 of Aurora B the AURKA bind to the AURKB binding protein INCENP, instead of TPX2 which is AURKA binding protein [10].

The Aurora B mitotic function is restored by the mutant Aurora A indicating that the binding to INCENP is important for AURKB’s unique functionality. Although AURKA needs G198 for TPX2 binding, and AURKB requires asparagine 142 for INCENP binding and function of AURKB [10].

Previous research has reported that certain compounds, including ZM447439 [17] and VX-680/MK-0457 [18] have demonstrated effects as Aurora kinase A inhibitors and Hesperidin [19] as AURKB. Despite significant experimental study on the interaction of inhibitors with AURKA and AURKB in different studies, decoding the atomic-level conformational changes of these two proteins due to inhibitor interactions is still crucial [20, 21].

With the rapid advancement of simulation and calculation methods [22], several molecular dynamics (MD) techniques, such as traditional MD [23, 24], multiple short molecular dynamics simulations [25, 26], accelerated MD (aMD) simulations [22, 27, 28], have been widely used to carry out target conformational evolution, various techniques for predicting binding free energy, including the Poisson Boltzmann surface area (MM-PBSA) method [29, 30], thermodynamics integration (TI) [31, 32], free energy perturbation (FEP) [33, 34], and solvated interaction energy (SIE) methods [35, 36], are frequently used to assess ligands’ capacity to bind to targets. Furthermore, techniques for machine learning and deep learning are presented to effectively examine the ligand-target binding process and reveal the underlying molecular causes of ligand-target interactions [37, 38]. These modeling techniques have also contributed to successful understandings of the inhibitor-receptor binding process.

**Fig 1:**
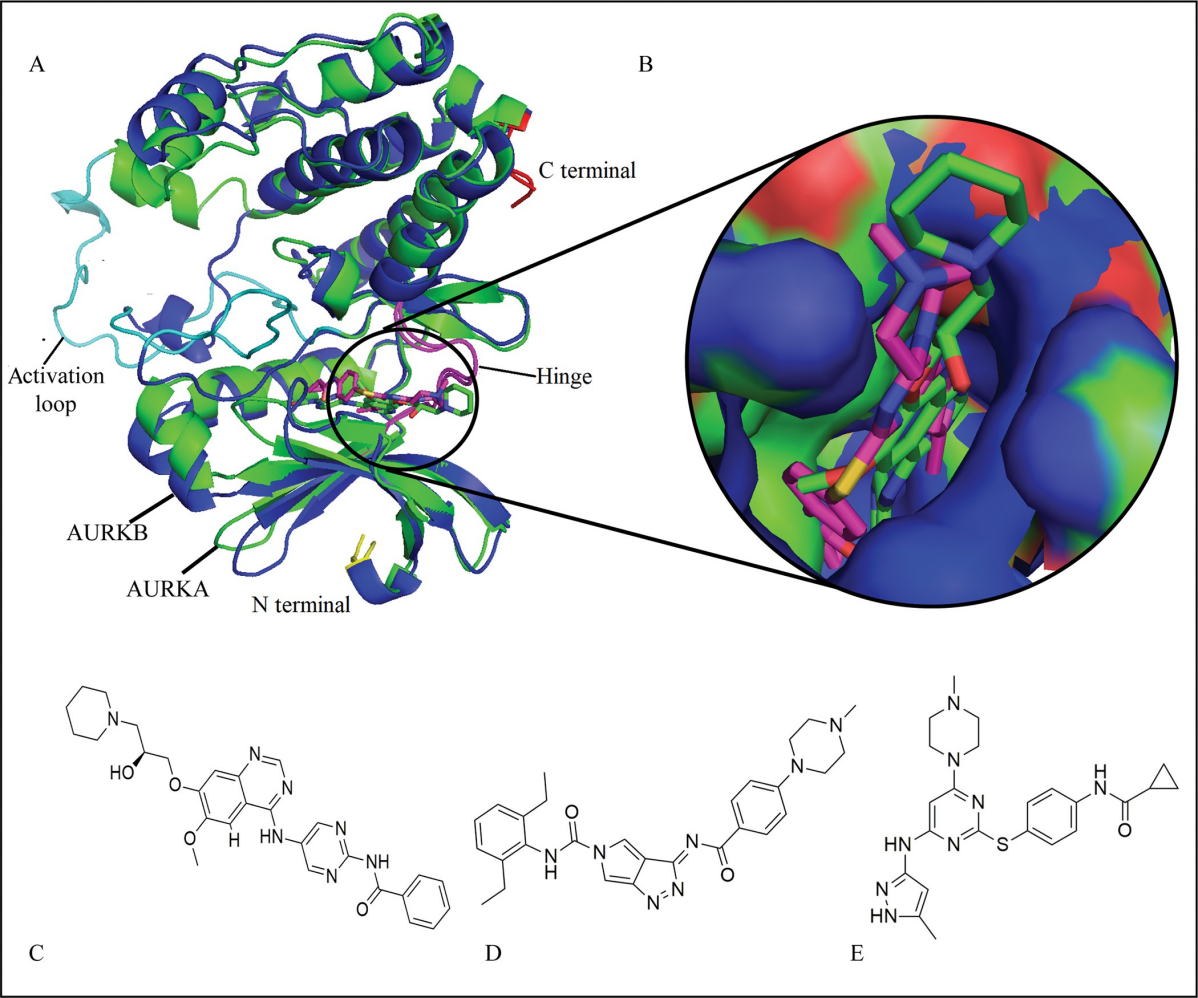
**(A)** Supxerposition of AURKA (green) and AURKB (blue) to analyze the conformation of bound inhibitors; **(B)** The plausible binding modes of inhibitors (represented with sticks) in AURKA and AURKB binding pocket (Shown in surface). **(C), (D),** and **(E)** represent the structures of HPM, MPY, and VX6, respectively.

The development of small drug like compounds that inhibit the activity of AURKs is still a major area of research. Two inhibitors MPY (2BMC) [39] and HPM (2C6E) [40] were designed to inhibit the activity of AURKA, while a small molecule VX6 (4AF3) [18] was developed to suppress the activity of AURKB. A crucial chemical process for the creation of small molecules that target AURKs can be found by further examining the differences in the binding patterns of VX6, HPM, and MPY to AURKA and AURKB. The plausible binding modes of these inhibitors are shown in Fig 1A and 1B, while the structures of HPM, MPY, and VX6 are shown in Fig 1C–1E. In this study, molecular dynamics simulations were used to enhance the conformational sampling of inhibitor-AURKs complexes, the cross-correlation matrix [41, 42] was used to understand the internal dynamics of inhibitor-bound AURKs, and calculations of residue-based free energy decomposition were used to identify the binding ability of VX6, HPM, and MPY to AURKs by employing MM/GBSA method. MM/GBSA offers a balanced approach, considering both molecular mechanics and solvation effects.

## 2. Material and methods

### 2.1. Simulated system setup

The crystal structures of AURKA complexed with HPM (PDB ID: 2BMC) and MPY (PDB ID: 2C6E) and AURKB complexed with VX6 (PDB ID: 4AF3) were retrieved from Protein Data Bank (PDB). The co-crystal poses of the inhibitors were extracted from the structures and six complexes i.e., HPM-AURKA, MPY-AURKA, VX6-AURKA, HPM-AURKB, MPY-AURKB, VX6-AURKB were prepared using the PyMOL software. During structure preparation, all the non-inhibitor molecules and crystal water were removed, and the missing hydrogen atoms were added using the Amber’s Leap tool. The simulation parameters of the proteins were prepared by using the ff14SB forcefield [43, 44]. Similarly, the geometries of the inhibitors were optimized at the semiempirical AM1 and Amber’s Antechamber module [45, 46], and Gasteiger changes were allocated to each inhibitor. The general amber forcefield (GAFF) [47] was used to generate the parameters for HPM, MPY, and VX6 inhibitors. After parametrization, the solvation of the complexes was done in a periodic box of 10 Å containing TIP3P water molecules [48]. Then systems were neutralized by the addition of 7 Cl^-^ ions in AURKA complexes and 8 Cl^-^ ions in AURKB complexes. The initial confirmation of the complexes was subjected to 250ns MD simulation with randomly assigned velocities.

### 2.2. Molecular dynamics simulations

Before subjecting the system to the production run, the prepared systems were optimized through steepest decent minimization of 10000 steps to remove the unfavorable atomic interactions. After that, the solvation system was equilibrated for an additional 10000 steps. Then the temperature of the system was gradually raised from 0 to 300K and then the systems were further optimized at 300K for equilibration. The systems that undergone the process of equilibration were then subjected to the production run for 250 ns long simulation at 310K temperature and 1 atm pressure using NPT ensemble. The SHAKE algorithm was used to constrain the hydrogen bond forming atoms, and the particle mesh Ewald (PME) approach was used to identify the long-range electrostatic interactions [49, 50] at the cutoff range of 10Å. The molecular dynamic simulations were run by using NAMD [51]. The molecular dynamic trajectories were analyzed using the VMD [52] and BIO3D package of R [53].

### 2.3. Principle component analysis

PCA has proven to be a valuable method for identifying coordinated motions in a collection of conformational structures obtained from either molecular simulations or experimental data. This technique has been widely used to study how changes in conformation affect the function of receptors [54, 55]. To perform PCA, we used the atomic coordinates obtained from the molecular dynamics simulations to construct the covariance matrix. This matrix is then diagonalized to produce a set of eigenvalues and eigenvectors. After diagonalization, the eigenvector of the matrix demonstrates the directions of movement of protein domains, and the correlated eigenvalues indicate the square mean fluctuations along the respective eigenvectors. The first few eigenvectors with high eigenvalues are very helpful in demonstrating the overall movements of proteins BIO3D package [53] of R was used to compute the dynamic movement of the protein complexes.

### 2.4. Calculations of MM/GBSA

MM/GBSA method provides more reliable binding free energy values than many molecular docking scoring functions [56, 57]. Similarly some studies have also shown that MM/GBSA approach is accurate and reliable enough for predicting the small drug like compounds and their protein targets binding free energies [58, 59]. Considering these studies in view, binding free energies of AURKs complexes were calculated using MM/GBSA method by employing the below mentioned equation.


$${\Delta G}_{bind}={\Delta G}_{comp}-{\Delta G}_{pro}-{\Delta G}_{lig}={\Delta E}_{ele}+{\Delta E}_{vdW}+{\Delta G}_{gb}+{\Delta G}_{nonpol}-T\Delta S$$

The ΔG_comp_, ΔG_pro_, and ΔG_lig_ indicate the binding energies of AURKs complexes. The ΔE_ele and_ ΔE_vdW_ show the electrostatic and van der Waals interactions of the inhibitors to AURKs. The term ΔG_gb_ presents the polar solvation energy which is solved by using the Generalized Born (GB) model [60] while ΔG_nonpol_ represent the nonpolar free energy terms. Lastly, TΔS indicates the entropy caused by the ligands.

## 3. Results and discussion

### 3.1. AURKA and AURKB’s structural fluctuation and flexibilities

**Fig 2:**
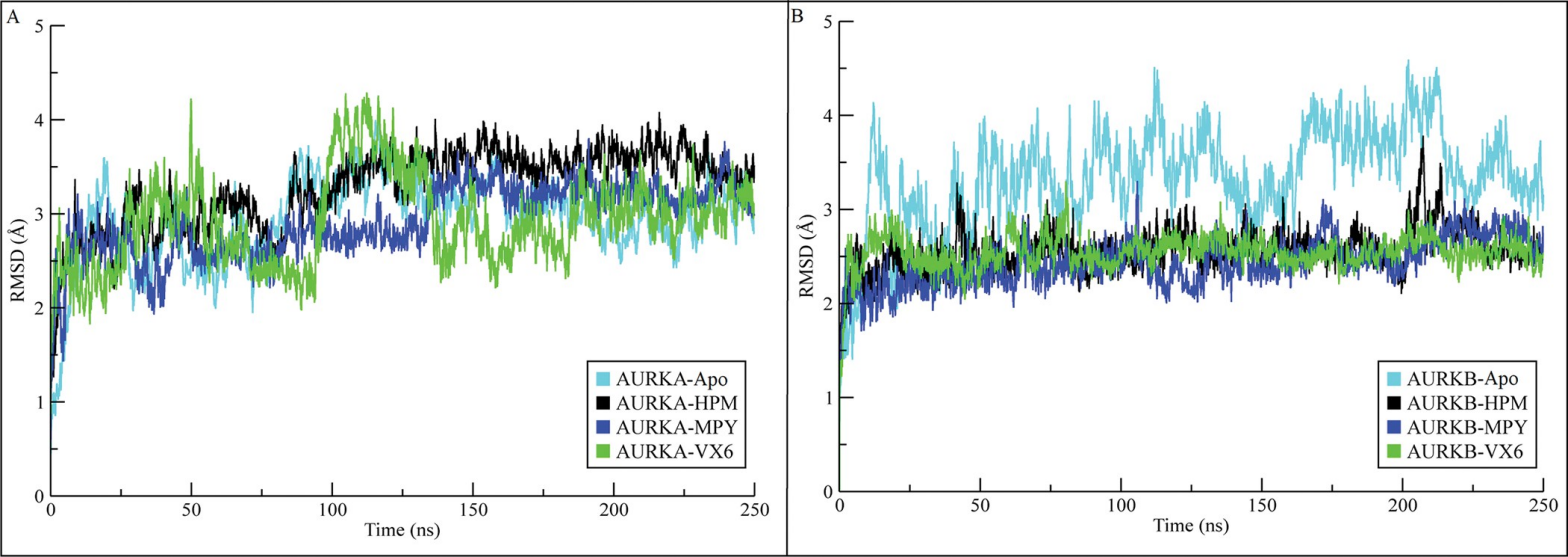
RMSDs of backbone atoms in AURKs complexes calculated during 250 ns simulation. (A) RMSD plots of AURKA complexes. (B) RMSD plots of AURKB complexes.

**Fig 3:**
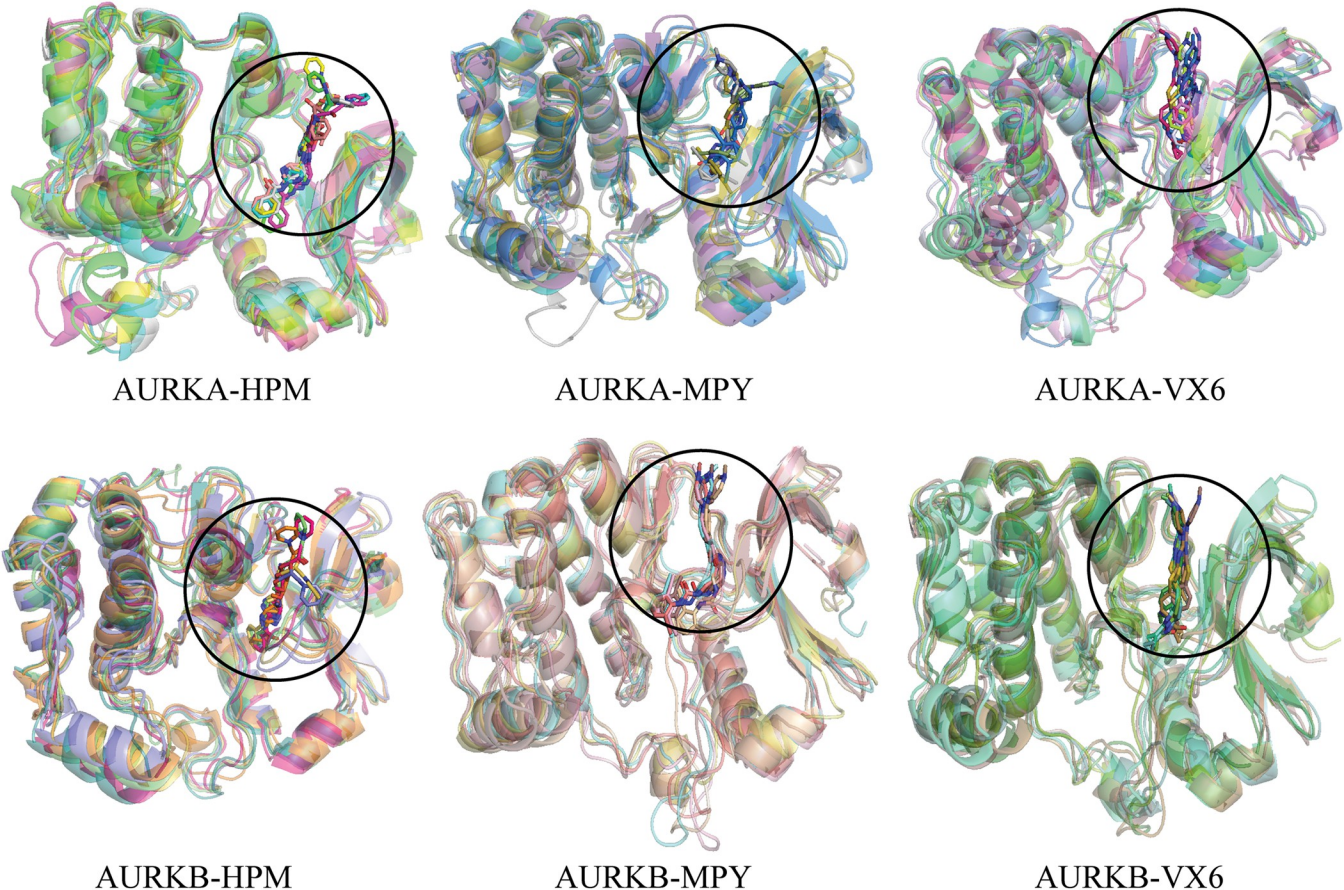
The superimposition of different snapshots of AURKA and AURKB complexes across the simulated time for conformational analysis.

Root mean square deviations (RMSDs) of the backbone atoms from the initial optimized configuration were calculated for apo structures of AURKA and AURKB and their complexes to evaluate the extent of structural fluctuations across molecular dynamics simulations (Fig 2A and 2B). The information from RMSD plots revealed that the AURKA systems attained equilibrium at 25 ns. After equilibration, the RMSD of the apo AURKA increased to ~3.5 Å at 100 ns and then attained a stable range of 3 Å at 150 ns. The RMSD of AURKA-HPM complex showed deviations to 2.5–3.5 Å till 100 ns and then attained stability at ~3.5 Å till the end of simulation. The RMSD of AURKA-MPY complex showed a similar trend to HPM complex whereas the RMSD of the AURKA-VX6 complex showed major deviation during 100 to 150 ns but it attained stability after 150 ns in the range of ~2.5-3Å. Among the three complexes, the AURKA-HPM showed higher deviations than the other two complexes. On the other hand, AURKB complexes attained stability at the start of the simulation and their RMSD values remained in the range of ~2–3 Å throughout the simulation time while the RMSD of apo structure was higher than the complexes as indicated in Fig 2B. The RMSD analysis showed that the systems attained equilibrium at specific value and remained stable during the simulation. Furthermore, the conformational changes in the AURKS structures were analyzed by aligning the different snapshots obtained at 0, 50, 100, 150, 200, and 250 ns. The alignment of the snapshots revealed that the ligands remained bound to the proteins pockets and did not dissociate during the simulation (Fig 3).

**Fig 4:**
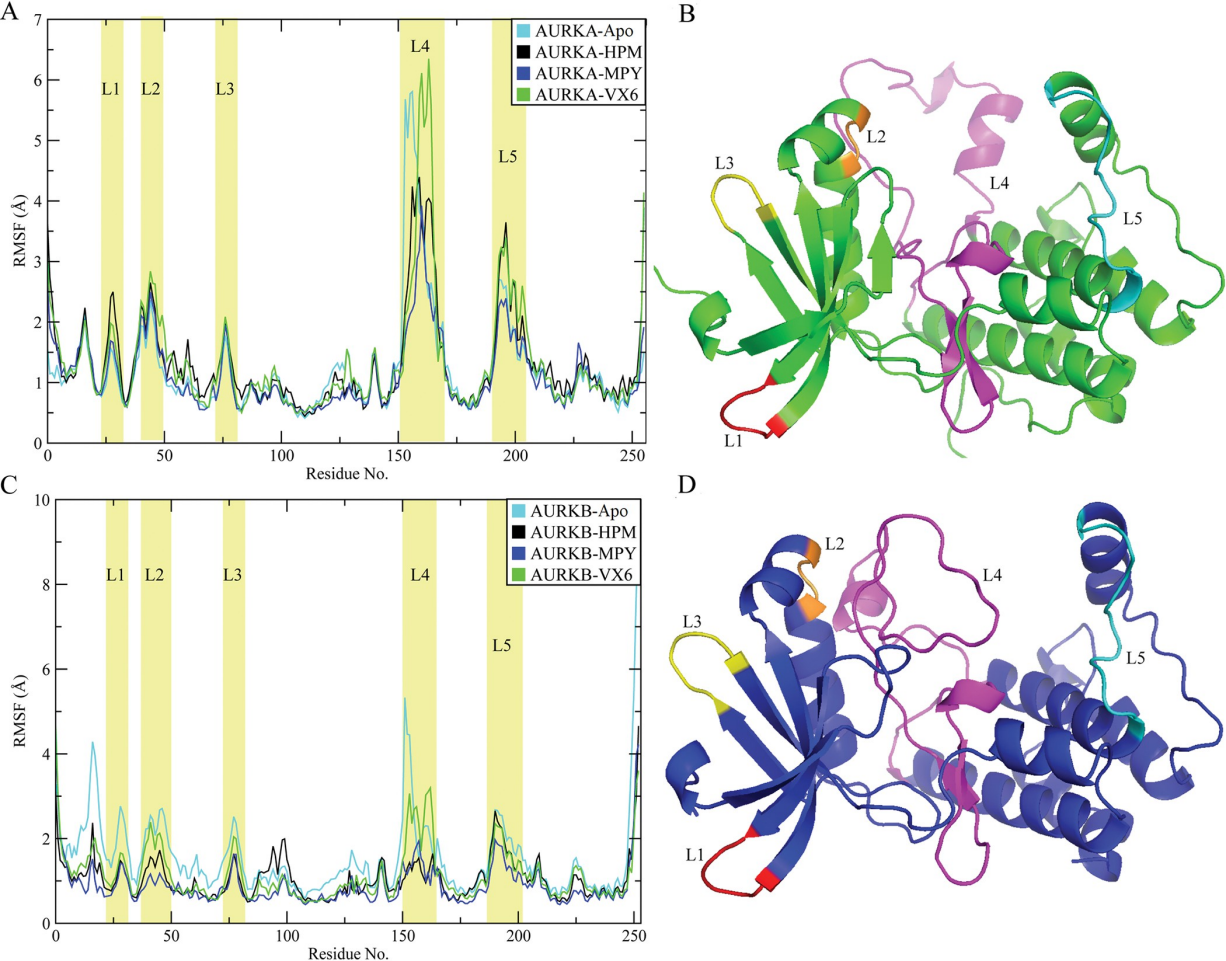
RMSF of residues in AURKA and AURKB during MD simulations: **(A)** RMSF for apo AURKA and its complexes with HPM, MPY, and VX6, **(B)** the structure of AURKA, **(C)** RMSF of apo AURKB and its complexes with three inhibitors and **(D)** the structure of AURKB.

To assess the fluctuations of AURKA and AURKB structures upon binding of HPM, MPY, and VX6 inhibitors, the Root Mean Square Fluctuations (RMSF) of the residues were calculated from the trajectories (Fig 4). The RMSF plots of AURKA and AURKB showed similar trends indicating that both structures have same number of rigid residues and flexible regions. Structural fluctuations were observed in five regions consisting of L1, L2, L3, L4 and L5 regions. These results showed that some residues from these regions were situated near the binding sites of the AURKA and AURKB. The RMSF values of AURKA and AURKB bound to VX6 were higher than the AURKs bound to the HPM and MPY, especially at the L2, L3, and L5 regions indicating that the binding of HPM and MPY restricted the motions of these regions. The structural analysis revealed that the regions L2 and L4 were near the binding sites of AURKA and AURKB which showed that some residues in these regions play a significant role in the binding selectivity of HMP, MPY, and VX6 towards the AURKs. While the residues in L1, L3 and L5 were not near the binding pocket and the fluctuations in these regions can play a vital role in the binding of these inhibitors to the AURKA and AURKB. The RMSF values of apo structures revealed that the flexibility in the loop regions was more in apo structure as compared to the complexes indicating the stability of protein structures upon binding of the ligands [61].

### 3.2. Dynamics behavior of AURKA and AURKB

**Fig 5:**
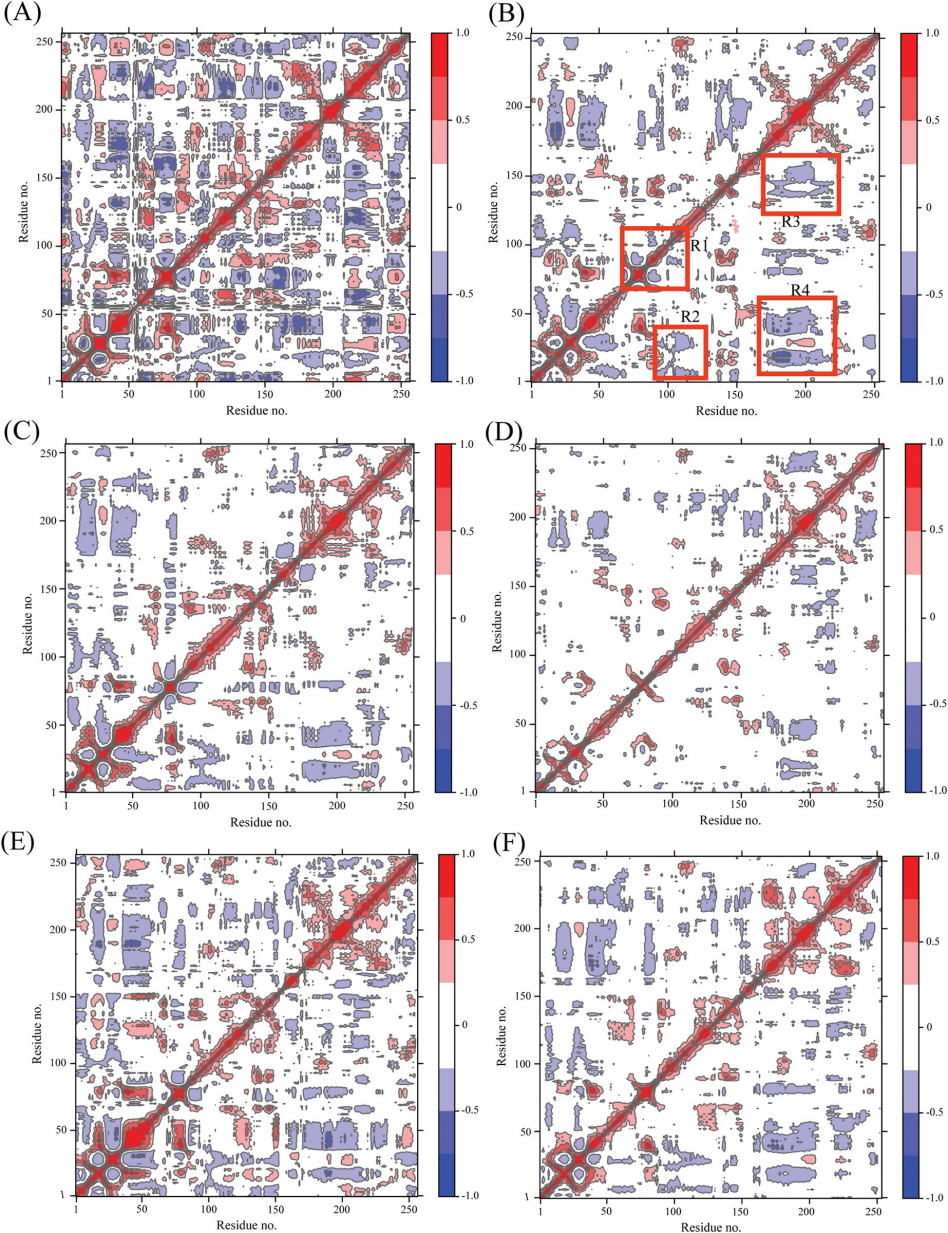
Cross correlation matrices derived from Cα atomic coordinates **(A), (C),** and **(E)** denoting AURKA with HPM, MPY, and VX6 respectively**; (B), (D),** and **(F)** depicting AURKB with HPM, MPY, and VX6.

The cross-correlation matrices of the Cα atomic coordinates of AURKA and AURKB complexes were computed to find the differences in structural dynamics (Fig 5). The red and pink colors show the positive correlated motions while blue and dark blue indicate the anti-correlated motions. The diagonal part of the matrix shows the correlated motion of domain relative to itself while the correlation in among different domains are depicted by the off-diagonal regions. As shown in Fig 5, the structural dynamics behavior of the AURKA and AURKB was influenced upon binding of the inhibitors, HPM, MPY, and VX6.

For AURKB complexes (Fig 5B, 5D and 5F) the anticorrelated motions were observed at three regions R2, R3, and R4, while the positively correlated motions were observed at the diagonal and R1 region. By comparing the HPM bound to AURKB, the binding of HPM to AURKA weakens the positive correlating motions at R1 regions and anticorrelated motions at the R2, R3, and R4 regions (Fig 5A). The binding of MPY to AURKA slightly affected the anti-correlated motions at R2, R3, and R4 regions while the binding of MPY to AURKB affected the anti-correlated motions but did not affect the positive correlated motions at R1 region (Fig 5D). By comparing the AURKA-VX6 and AURKB-VX6, the associations did not alter the motions at R1 region in AURKA, but it strengthened the anticorrelated motions in R2, R3, and R4 regions in AURKA (Fig 5E). According to the above discussion, the binding of identical inhibitors to AURKA and AURKB lead to motion mode difference, indicating that the residues in R1–R4 regions may be involved in binding with HPM, MPY and VX6 [62].

### 3.3. PCA analysis

**Fig 6:**
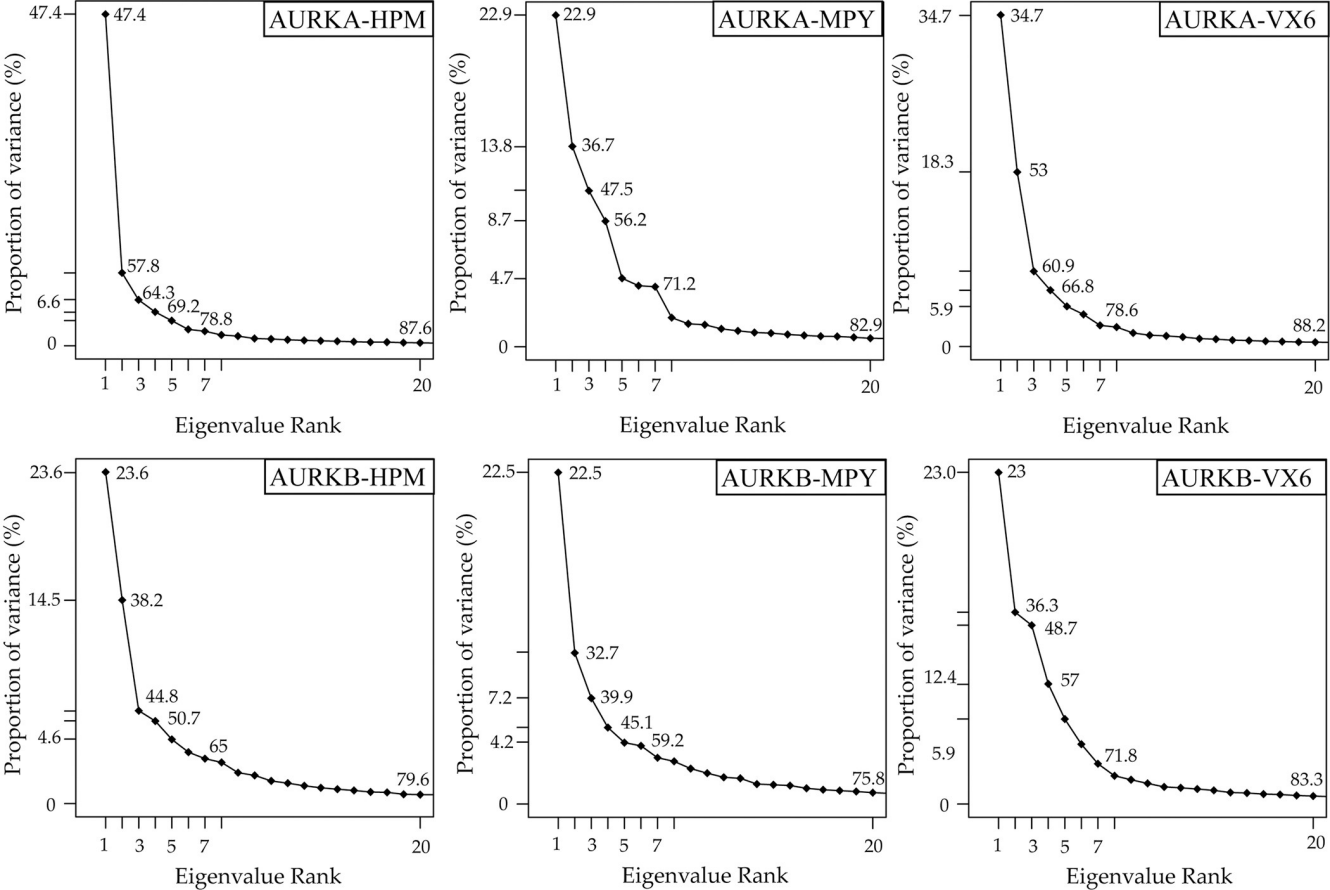
The percentage proportion of variance of AURKA and AURKB bound to HPM, MPY, and VX6.

The use of principal component analysis (PCA) is widespread in the investigation of concerted movements of protein structural domains. This approach can effectively filter significant collective motions from structural ensembles obtained from experimental or simulation studies. In this study, PCA was utilized to decode the molecular mechanism underlying the binding selectivity of HPM, MPY, and VX6 to AURKA and AURKB. To perform PCA, a covariance matrix was constructed using the Cα atomic coordinates extracted from the starting conformational trajectory (SCT). The proportion of variance of first eigenvalue for the HPM bound to AURKA was 47.4% that was higher than the first eigenvalue of AURKB bound to HPM, indicating the higher structural variation in AURKA-HPM complex (Fig 6A and 6D). The binding of MPY to AURKA and AURKB did not show major difference in the variance of the structures as the eigenvalues for AURKA and AURKB were 22.9% and 22.5%, respectively (Fig 6B and 6E). Lastly, the binding of VX6 showed more variation in AURKA than AURKB. The results indicated that the binding of these inhibitors influence the structural variations of the AURKA and AURKB.

### 3.4. Binding free energy calculations

The MM/GBSA approach was utilized to determine the binding free energies of HPM, MPY, and VX6 to AURKA and AURKB. This involved calculating the energetic data for three hundred structural frames obtained from a 250 ns trajectory at 2 ns intervals. The results of the MM/GBSA calculation for all three ligands bound to AUKRA and AURKB are presented in Table 1.

**Table 1:** Binding affinities of inhibitors to AURKA and AURKB calculated with MM/GBSA Approach ^a^.

	HPM-AURKA		HPM-AURKB		MPY-AURKA		MPY-AURKB		VX6-AURKA		VX6-AURKB	
	Mean	SEM^b^	Mean	SEM^b^	Mean	SEM^b^	Mean	SEM^b^	Mean	SEM^b^	Mean	SEM^b^
**ΔE**_**vdw**_	-69.82	0.22	-87.48	0.45	-63.31	0.28	-69.43	0.50	-61.01	0.35	-69.74	0.37
**ΔE**_**ele**_	-0.01	0.42	-7.59	0.43	-2.37	0.15	-1.36	0.14	-4.17	0.19	-3.92	0.18
**ΔE**_**GB**_	23.76	0.44	34.71	0.41	22.77	0.18	22.37	0.20	18.83	0.23	20.81	0.21
**ΔE**_**surf**_	-7.02	0.02	-8.98	0.02	-6.22	0.02	-6.86	0.02	-5.82	0.02	-6.47	0.02
**ΔG**_**gas**_	-69.83	0.67	-95.08	0.50	-65.88	0.33	-70.80	0.54	-65.19	0.37	-73.66	0.43
**ΔG**_**solv**_	16.74	0.44	25.73	0.41	16.54	0.17	15.51	0.19	13.00	0.22	14.33	0.21
**TΔS**	-23.98	2.02	-21.83	1.47	-23.68	1.36	-28.07	1.25	-21.64	1.96	-20.10	1.56
**ΔG**_**total**_	-53.09	0.43	-69.34	0.33	-49.34	0.32	-55.28	0.49	-52.18	0.33	-59.32	0.42

a, unit of energy components are in kcal/mol.

b, Standard Error of Mean.

**Fig 7:**
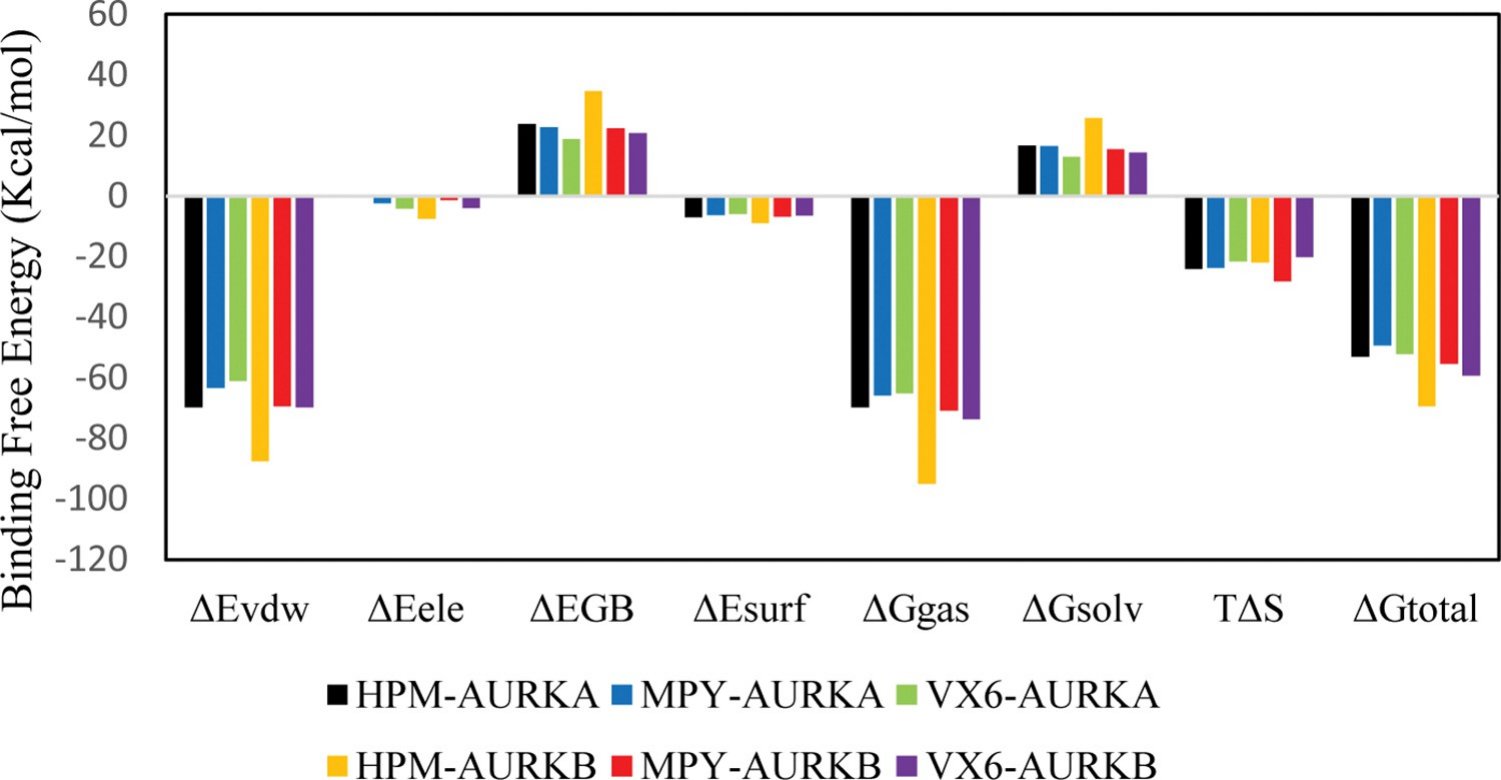
The relative contribution of binding energy terms for AURKA and AURKB complex calculated using MM/GBSA method.

The ΔE_vdW_ value for AURKB-HPM was higher than AUKRA-HPM complex. The similar trend was observed in the ΔE_vdW_ value of MPY and VX6 complexes. The ΔE_ele_ energy component for AURKA-HPM was -0.01±0.42, while it was -7.59±0.43 for HPM-AURKB complex. The electrostatic energy contributions indicated that the AURKA complexes showed better electrostatic energies than AURKB complexes except for HPM complexes. After analyzing the surface energies, the AURKB-HPM complex showed a better surface energy of -8.98±0.02 than all other complexes. The ΔG_total_ values of HPM-AUKRA (-53.09±0.09), HPM-AURKB (-69.34±0.33), MPY-AURKA (-49.34±0.32), MPY-AURKB (-55.28±0.49), VX6-AURKA (-52.79±0.49), VX6-AURKB (-59.32±0.42) indicated that HPM had better selectivity towards AURKB rather than AURKA. Similarly, MPY and VX6 had better binding free energies with AURKB than AURKA. The relative contribution of energy terms in the AURKs complexes is shown in Fig 7.

### 3.5. Binding selectivity uncovered by inhibitor-residue interaction analyses

**Fig 8:**
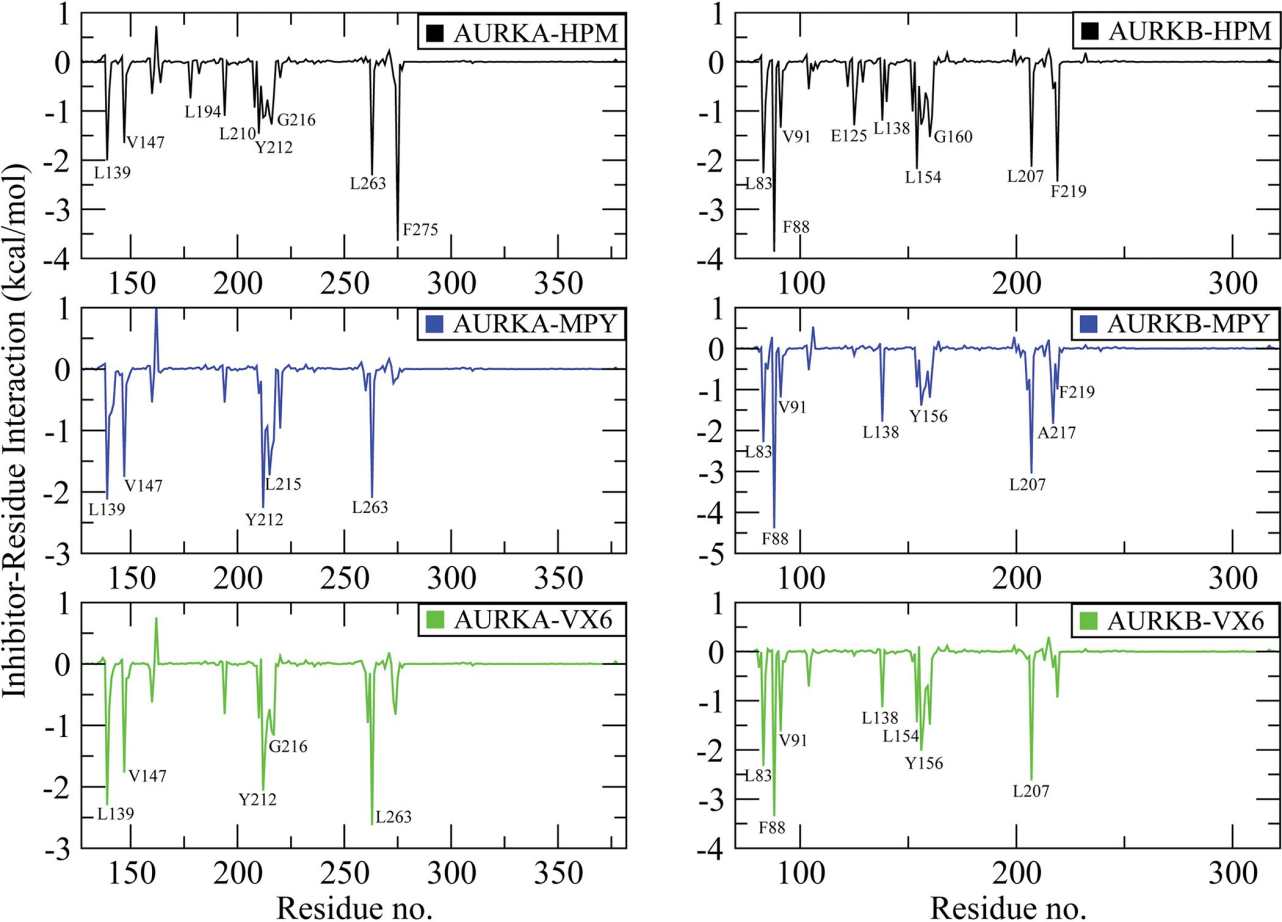
Residues stronger than -1 kcal/mol are specified for inhibitor-residue interactions determined using the residue-based free energy decomposition method. **(A)** the AURKA-HPM complex**, (B)** AURKB-HPM complex**, (C)** AURKA-MPY complex, **(D)** AURKB-MPY complex, **(E)** AURKA-VX6 complex and **(F)** AURKB-VX6 complex.

To clarify the binding selectivity of the three inhibitors (HPM, MPY, and VX6) to AURKA and AURKB, we used the MM/GBSA method to analyze the interactions between the inhibitors and specific residues. Table 2 Demonstrate the decomposition of ΔGligand–residue values into contributions from the sidechain and backbone of key residues in AURKA and AURKB bound by each inhibitor. We discovered that the sidechains of residues play an important role in contributing energy to inhibitor-residue interactions. Fig 8 displays the key residues of AURKA and AURKB that form important inhibitor–residue interactions with energies stronger than -1 kcal/mol [63]. Additionally, to determine hydrogen bond interactions (HBIs) between the inhibitors and AUKRA, and AURKB, we used the CPPTRAJ tool in Amber 21. The results are summarized in Table 3.

**Table 2:** Interactions between important AURKA and AURKB residues and three inhibitors (all values in kcal/mol).

			Inhibitor							
			HPM			MPY			VX6	
AURKs	Key residues	Total	Sidechain	Backbone	Total	Sidechain	Backbone	Total	Sidechain	Backbone
AURKA	L139	-2.00	-2.12	0.12	-2.12	-1.96	-0.16	-2.29	-2.54	0.25
	V147	-1.65	-1.79	0.14	-1.75	-1.68	-0.07	-1.76	-1.70	-0.06
	L194	-1.09	-1.16	0.07	-0.54	-0.59	0.05	-0.81	-0.80	-0.03
	L210	-1.46	-1.44	-0.02	-0.46	-0.39	-0.07	-0.88	-0.76	-0.12
	Y212	-1.13	-1.28	0.15	-2.25	-2.27	0.02	-2.06	-1.91	-0.15
	L215	-1.07	-0.63	-0.44	-1.72	-1.00	-0.72	-0.73	-0.30	-0.43
	G216	-1.27	-0.72	-0.55	-1.31	-0.71	-0.60	-1.08	-0.61	-0.47
	L263	-2.30	-2.50	0.20	-2.09	-2.19	0.10	-2.62	-2.63	0.01
	F275	-3.63	-3.37	-0.26	-0.14	-0.15	0.01	-0.22	-0.19	-0.03
AURKB	L83	-2.26	-2.36	0.10	-2.28	-2.19	-0.09	-2.32	-2.53	0.21
	F88	-3.86	-3.48	-0.38	-4.38	-4.06	-0.32	-3.34	-3.17	-0.17
	V91	-1.33	-1.34	0.005	-1.19	-1.28	0.09	-1.61	-1.57	-0.04
	E125	-1.29	-1.08	-0.21	-0.16	-0.15	-0.01	-0.08	-0.06	-0.02
	L138	-1.19	-1.21	0.02	-1.78	-1.89	0.10	-1.12	-1.15	0.03
	L154	-2.17	-2.18	0.01	-0.94	-0.90	-0.04	-1.43	-1.24	-0.19
	Y156	-1.27	-1.42	0.15	-1.38	-1.55	0.17	-2.01	-1.94	-0.07
	G160	-1.52	-0.70	-0.82	-1.19	-0.57	-0.62	-1.48	-0.67	-0.81
	L207	-2.13	-2.35	0.22	-3.04	-2.80	-0.24	-2.61	-2.65	0.04
	F219	-2.43	-2.36	-0.07	-0.99	-0.94	-0.05	-0.93	-0.91	-0.02

**Table 3:** Hydrogen bonding interactions between inhibitors and AURKA/B computed by the CPPTRAJ.

Complexes	Hydrogen Bonds	Distance (Å)	Angle ^(^°)	Occupancy ^(^%)
HPM-AURKA	Ala87-H-----HPM-N17	3.25	144.00	78.23
HPM-AURKB	Lys38-HE2-----HPM-O36	3.29	138.31	34.32
MPY-AURKA	Asp148-HA-----MPY-O26	3.55	138.44	44.09
MPY-AURKB	Gly91-CA-----MPY-C24	3.70	148.61	70.51
VX6-AURKA	Ala87-H-----VX6-N14	3.67	146.61	64.48
VX6-AURKB	Ala88-H-----VX6-N14	3.64	148.54	74.39

For the HPM bound to AURKA and AURKB, HPM showed better interactions with L139, V147, L194, L210, Y212, G216, L263, and F275 in AURKA and all the interactions were stronger than -1kcal/mol (Fig 8A). Additionally, a Hydrogen bond interaction (Ala87-H——HPM-N17) was detected with an occupancy of 78.23% (Table 3). The HBI indicated that the hydrogen atom of Ala87 residues engaged in hydrogen bonding with the nitrogen seventeen of HPM. On comparison with the binding of HPM to AURKB, it was observed that the binding modes of HPM with AURKB were like AURKA (Fig 8B). It was observed that the interactions difference of HPM with residues (L139, L83), (V147, V91), (L194, L138), (L210, L154), (G216, G160), (L263, L207) corresponding to AURKA and AURKB was less than 0.30 kcal/mol. The interaction energy difference of HPM with F275 in AURKA and F219 in AURKB was strengthened by 1.20 kcal/mol, indicating that these residues play a significant role in binding selectivity of HPM to AURKA over AURKB.

In case of MPY binding to the AURKA, the interactions stronger than -1.00 kcal/mol were observed in the residues L139, V147, Y212, L215, and L263 (Fig 8C). The hydrogen bond interactions showed that the HA atom of Asp148 engaged in hydrogen bonding with the O36 of MPY with an occupancy of 44.09%. The binding mode of MPY in AURKB was like AURKA as the interacting residues (L139, L83), (V147, V91), (Y212, Y156), and (L263, L207) depicted the better interactions with energy stronger than -1.00 kcal/mol. The binding interactions of L207 and Y212 were strengthened by 0.95kcal/mol and 0.87kcal/mol, indicating these residues were involved in the binding selectivity of MPY towards AURKA and AURKB [64].

**Fig 9:**
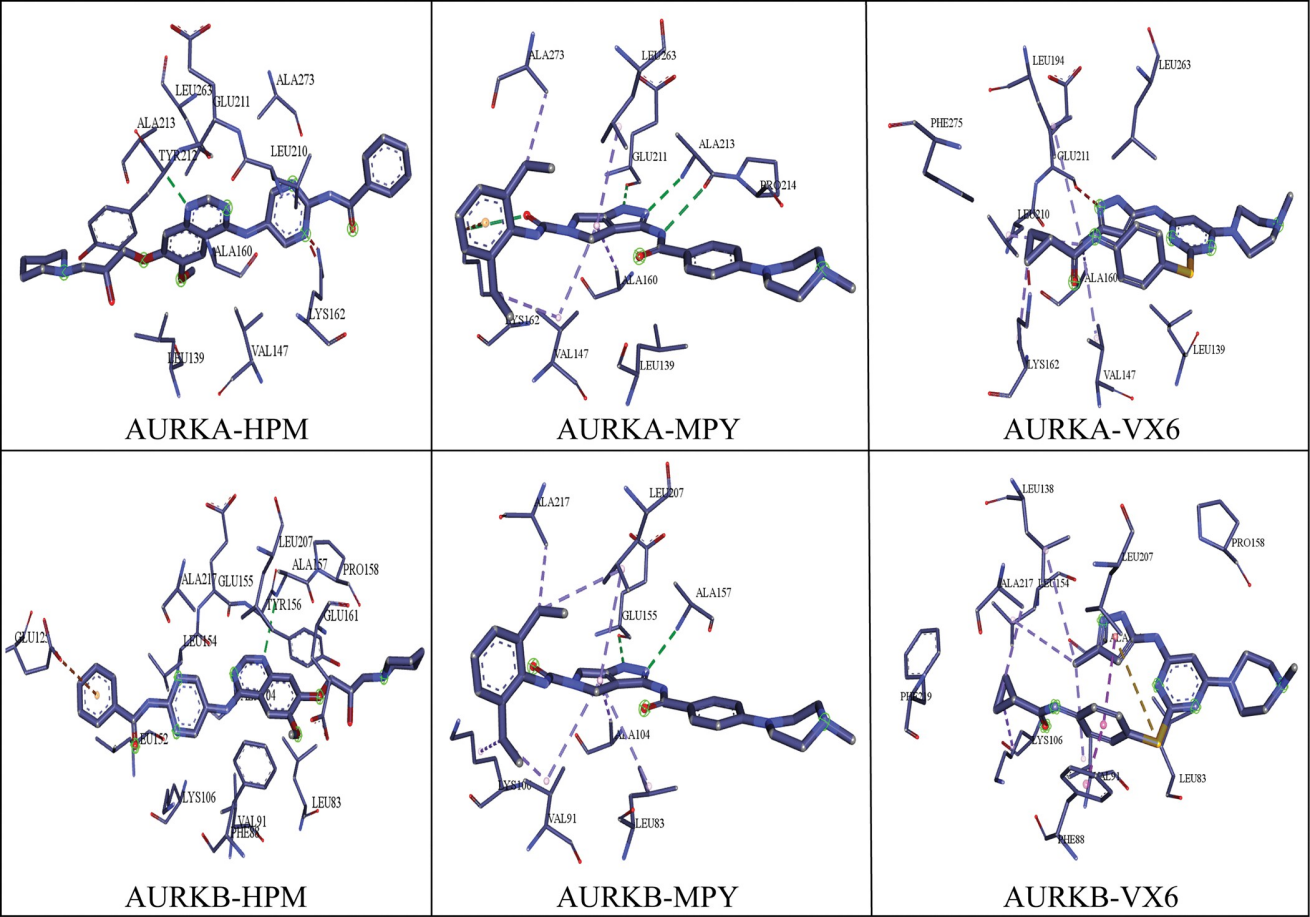
The binding modes of the inhibitors with the interacting residues of AURKA and AURKB proteins.

Lastly, VX6 bound to AURKA and AURKB interacted with four residues, (L139, L83), (V147, V91), (Y212, Y156), and (L263, L207) as show in (Fig 8E and 8F). VX6 formed HBI with AURKA (Ala87-H-----VX6-N14), and AURKB (Ala88-H-----VX6-N14) with an occupancy of 64.48% and 74.69%, respectively. The interactions of VX6 with L139 and L263 in AURKA were strengthened by -2.29 and -2.32kcal/mol as compared to the binding of VX6 to L83 and L207 with AURKB, which showed that these residues are important for the binding of VX6 to AURKA and AURKB. The binding modes of the inhibitors with the interacting residues are shown in Fig 9.

**Fig 10:**
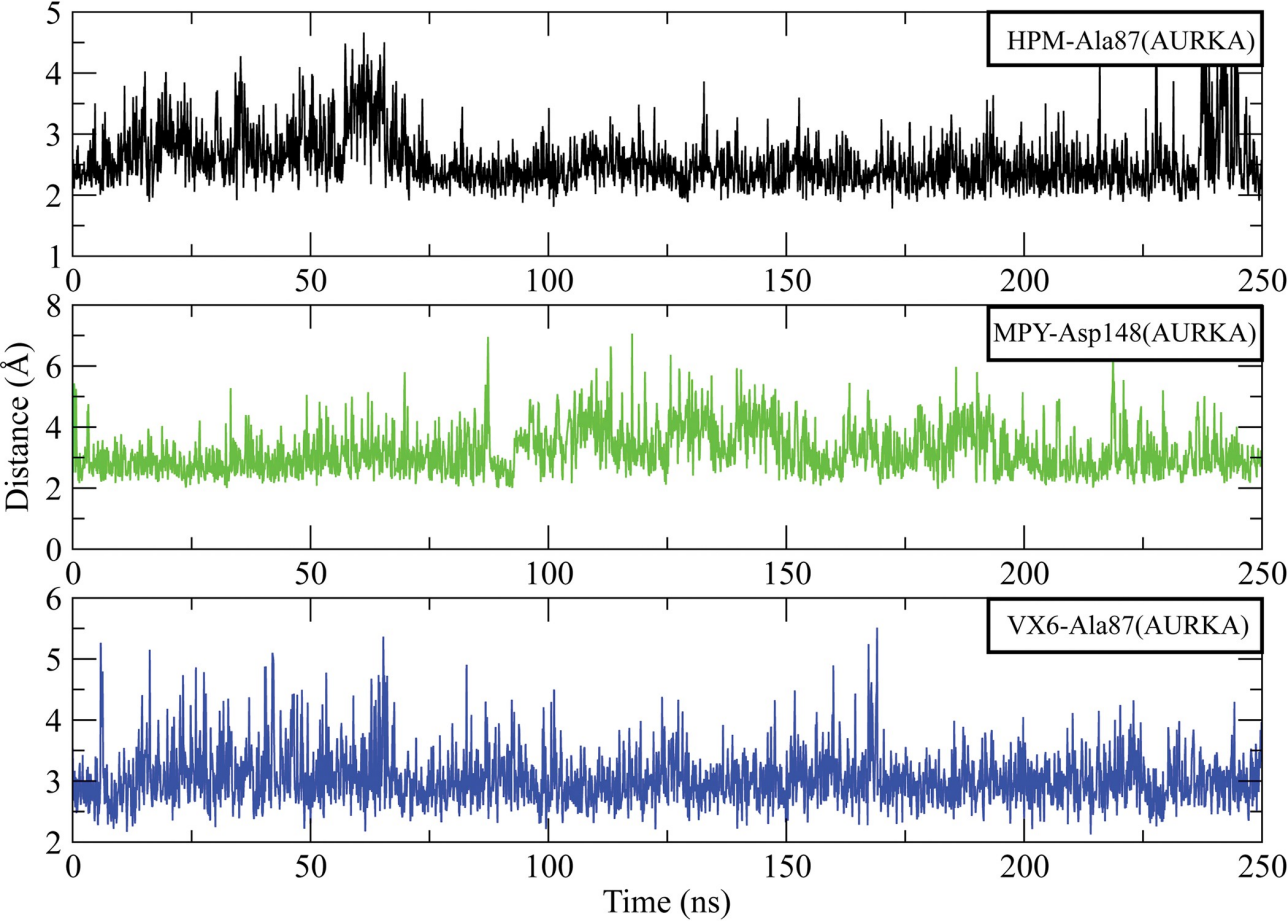
The distance plots of hydrogen bond forming residues of AURKA with HPM, MPY and VX6 inhibitors for entire simulation time.

**Fig 11:**
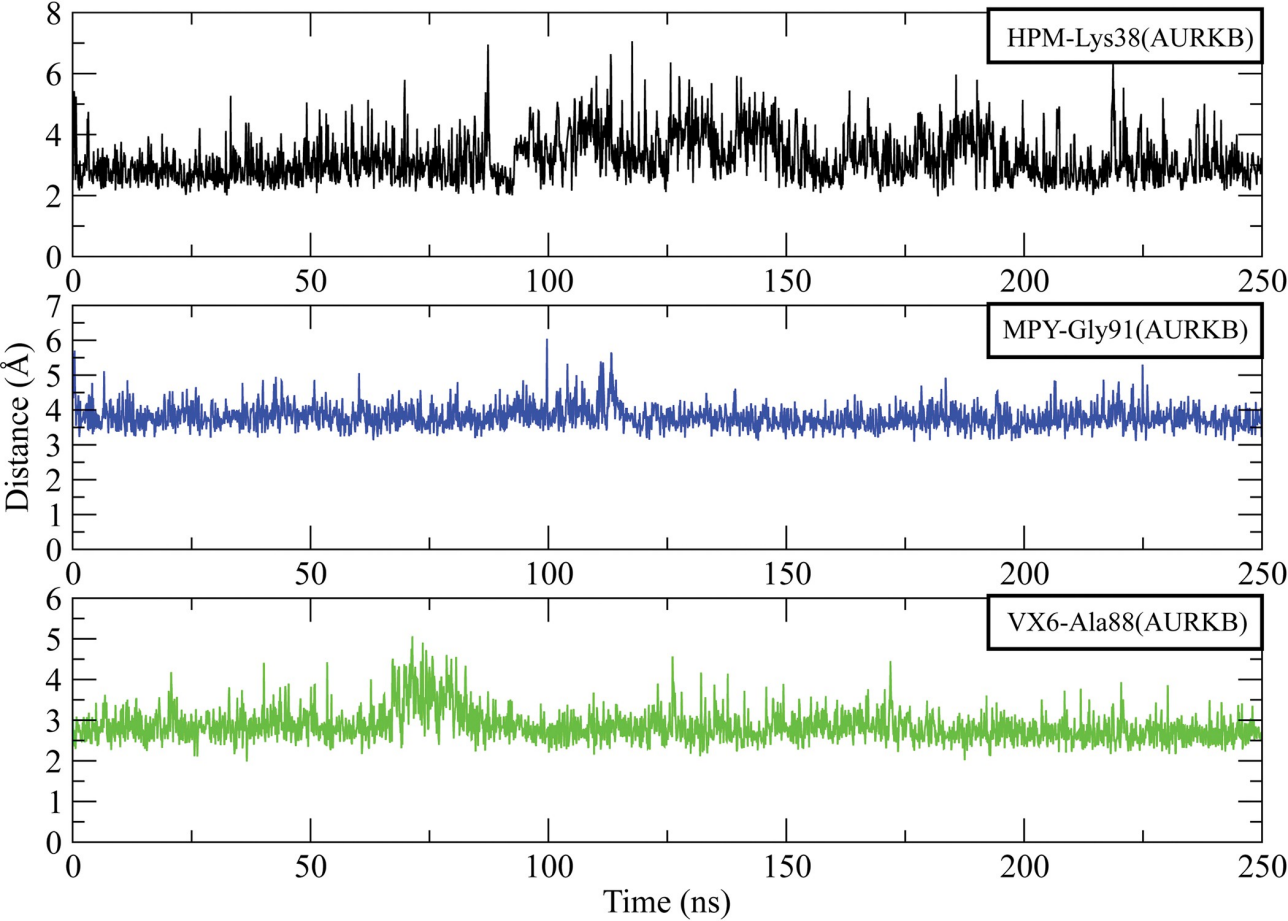
The distance plot of hydrogen bond forming residues of AURKB with HPM, MPY and VX6 inhibitors for entire simulation time.

Further, the distances between the hydrogen bond forming residues with the inhibitors were calculated to analyze the stability of the complexes. The analysis of AURKA complexes showed that the distance between HPM and Ala87 at the start of simulation was 3.25 Å which gradually increased to 4.5 Å at 60 ns but it dropped to 2.5 Å at 75 ns and then remained in this range till 225 ns. The distance increased to 5 Å towards the end of simulation. Similarly, the average distance between MPY and Asp148 3.5 Å during 250 ns simulation. The distance between VX6 and Ala87 was 3.5 Å in most of the frames while in some frames it reached 5 Å (Fig 10). On the other hand, the distance between HPM and Lys38 of AURKB was in the range of 3.5 Å most of the time. The average distance between MPY and Gly91 was 4 Å and the distance between VX6 and Ala88 was 3 Å (Fig 11).

The role of residual contribution in the total binding free energy was further estimated by alanine scanning. Table 2 indicates that F275 play an important role in binding free energy of HPM-AURKA complex. In MPY-AUKRA complex, Y212 showed better contribution in binding free energy while in VX6-AURKA complex, the major energy contribution was exhibited by L263. In the AURKB complexes, F88 showed major contributions in all complexes. These residues were mutated to alanine and then their effect on total binding free energy was calculated by alanine scanning. The reduction in the binding free energies upon mutation is shown in Table 4. The mutation of F275 to alanine in HPM-AURKA complex reduced the binding free energy to -49.07 with an energy loss of -4.01 kcal/mol. The mutation of Y212 in MPY-AURKA caused an energy loss of -2.23 kcal/mol while the mutation of L263 in VX6-AURKA complex resulted in an energy loss of -4.73 kcal/mol. In AURKB complexes, the mutation of F88 to alanine showed the more energy loss in HPM-AURKB complex with a value of -4.07 kcal/mol. The loss of energy due to mutation in these residues indicated the significance of these residues in total binding free energy of complexes.

**Table 4:** Binding free energies of the complexes upon mutation of residues to alanine.

	HPM-AURKA		HPM-AURKB		MPY-AURKA		MPY-AURKB		VX6-AURKA		VX6-AURKB	
	F275A		F88A		Y212A		F88A		L263A		F88A	
	Mean	SEM	Mean	SEM	Mean	SEM	Mean	SEM	Mean	SEM	Mean	SEM
**ΔE**_**vdw**_	-66.25	0.38	-84.36	0.42	-61.50	0.28	-66.57	0.50	-55.94	0.33	-66.18	0.35
**ΔE**_**ele**_	0.18	0.43	-7.53	0.44	-2.18	0.15	-1.35	0.13	-4.21	0.19	-3.74	0.18
**ΔE**_**GB**_	23.84	0.46	35.54	0.40	22.76	0.18	22.83	0.20	18.18	0.23	20.98	0.22
**ΔE**_**surf**_	-6.86	0.02	-8.91	0.02	-6.17	0.02	-6.65	0.02	-5.47	0.02	-6.37	0.02
**ΔG**_**gas**_	-66.06	0.63	-91.89	0.49	-63.69	0.33	-67.93	0.55	-60.15	0.35	-69.93	0.40
**ΔG**_**solv**_	16.98	0.46	26.62	0.40	16.58	0.18	16.17	0.20	12.70	0.22	14.60	0.22
**ΔG**_**total**_	-49.07	0.39	-65.27	0.29	-47.10	0.31	-51.75	0.49	-47.44	0.30	-55.32	0.40
**ΔΔG**_**bind**_	-4.01	0.99	-4.07	1.18	-2.23	0.39	-3.52	0.93	-4.73	0.63	-3.99	1.04

**ΔΔG**_**bind**_ **=** Difference in mutant and wild type energy values.

## 4. Conclusion

Molecular dynamics simulations were conducted on three AURKA and AURKB systems bound by three inhibitors (HPM, MPY, and VX6) to gain insights into their binding selectivity for anti-cancer drug development. The study revealed that the structural flexibility of two regions L4 and L5 in AURKA was higher than that of AURKB, and these domains exhibited distinct internal dynamics behavior. MM/GBSA calculations showed that the binding free energies of HPM, MPY, and VX6 with AURKB were higher than the AURKA complexes, indicating the superior selectivity and binding ability of inhibitors towards AURKB. Furthermore, residue-based free energy decomposition analysis identified four common residue pairs (L139, L83), (V147, V91), (L210, L154), and (L263, L207) that played a significant role in inhibitor binding affinity, suggesting their crucial role in determining the selectivity of inhibitors towards AURKA and AURKB. These findings could provide a better understanding of the molecular mechanisms and structure-affinity relationships for designing highly selective AURKs inhibitors.
